# The mid-long term results of reconstructional cage and morselized allografts combined application for the Paprosky type III acetabular bone defects in revision hip arthroplasty

**DOI:** 10.1186/s12891-019-2915-3

**Published:** 2019-11-07

**Authors:** Qiang Xiao, Haoyang Wang, Kai Zhou, Duan Wang, Tingxian Ling, Fuxing Pei, Zongke Zhou

**Affiliations:** 1 Department of Orthopaedics, Chengdu Second People’s Hospital, Chengdu, People’s Republic of China; 2 Department of Orthopaedics, West China Hospital, Sichuan University, 37# Wuhou Guoxue Road, Chengdu, 610041 People’s Republic of China

**Keywords:** Revision hip arthroplasty, Reconstructional cage, Morselized allografts, Acetabular bone defects

## Abstract

**Background:**

Severe acetabular bone defects is a complex problem in revision hip arthroplasty, cage is one of the reconstruction options. The purpose of this study is to report the mid-long term clinical and radiographic results of Paprosky type III acetabular bone defects revised with reconstructional cage and morselized allogeneic cancellous bone graft without impaction.

**Methods:**

We retrospectively analyzed 28 patients who underwent revision hip arthroplasty with reconstructional cage and allogeneic cancellous bone graft between January 2007 and January 2016. There were 13 Paprosky type IIIA bone defect patients and 15 Paprosky type IIIB bone defect patients and 4 patients of the 15 were also with pelvic discontinuity. Clinical assessment included Harris Hip Score (HHS) and Short Form-12 (SF-12). Radiographic assessment included center of rotation, cage migration, and bone graft incorporation.

**Results:**

All patients were followed up with a mean follow-up of 79.5 months (range 38–141), HHS improved from 31.4 (13–43) points preoperatively to 84.6 (55–94) points at last follow-up and SF-12 also improved significantly. There was 1 re-revision for the cage loosening and screw breakage at 61 months after surgery, and 2 patients had nonprogressive radiolucency in zone III and the junction of zone II and zone III at the bone implant interface.

**Conclusion:**

The reconstructional cage combining with morselized allografts without impaction achieves a good result with a high complete allograft incorporation rate in Paprosky type III acetabular bone defects.

## Introduction

In revision hip arthroplasty, severe acetabular bone defect is a complex problem with the goals of achieving stable and durable fixation of the acetabular component, restoring acetabular bone stock and reconstructing the hip rotation center [1]. There are several reconstruction options to choose, including impaction bone grafting and cemented cup [2], hemispheric acetabular component [3, 4], porous metal augments [5–7], ring and reconstruction cage [8, 9], oblong components [10], cup-cage reconstruction [11–13], and custom triflange implants [14, 15].

The porous hemispherical components provide structure for the bone ingrowth in order to achieve firmly fixation and have satisfactory follow-up results [5–7]. But when the acetabular bone defect is severe, placing the acetabular components to anatomical position and simultaneously achieving stable fixation may be difficult. In this situation, reconstructional cage is an alternative option [16].

Previous reports of reconstructional cage show a good mid to long-term results in acetabular revision arthroplasty [8, 9, 17, 18]. When combining with bone allografts, cage can bridge the bone defect to protect the underlying allograft during the bone remodeling phase. This may contribute to restoring acetabular bone stock and further revision surgery [19]. However, these studies mixed the results of Paprosky type II and III bone defects [8, 17, 18], and the result of reconstructional cage in Paprosky type III bone defects was not very clear.

Gamma irradiation is wildly used for allograft sterilization in tissue banks, and its effectiveness and safety has been confirmed. But it can result in a decrement in the mechanical strength of the allograft and affect the biological performance of allograft [20]. Povidone-iodine has good sterilization ability, and it has advantages in maintaining the tissue viability of allograft when used for allograft sterilization [21–23]. At the same time, the process of allograft sterilization with povidone-iodine is relatively simple comparing with gamma irradiation. Therefore povidone-iodine may be an alternative option for allograft sterilization.

The purpose of this study is to report the mid-long term clinical and radiographic results of using reconstructional cage and morselized allogeneic cancellous bone graft without impaction for Paprosky type III acetabular bone defects in acetabular revision and introduce our experience in bone allografts sterilization.

## Methods and patients

This study was approved by the Ethics Committee of our institution. We systematically searched the patients whose diagnosis included acetabular bone defects in the joint replacement registration system of our hospital from January 2006 to January 2016. There were 158 patients diagnosed with type III acetabular bone defects according to the Paprosky classification [24], 28 of whom underwent revision hip arthroplasty with reconstructional cage and morselized allogeneic cancellous bone graft. The preoperative and postoperative clinical and radiographic examinations and surgical data of these 28 patients were available.

Twenty-eight patients had 28 revision hips. Thirteen patients had a type IIIA bone defect and 15 patients had a type IIIB bone defect (4 of these 15 patients had pelvic discontinuity). The type of the acetabular defects was determined by preoperative radiographic examination and intraoperative assessments. There were 13 males and 15 females whose average age at revision was 56.4 years (range 36–75) and their average body mass index was 23.9 kg/m2 (range 18.3–29.6). Twenty-three had left hip involvement and 5 had right hip involvement. Twenty-one were total hip revision and 7 were acetabular revision, and 5 had primary total hip replacement or hip revision on the opposite side. The main cause of revision was aseptic loosening (AL) including 25 patients and 3 of periprosthetic infection. The initial diagnosis of these patients was osteoarthritis in 23 cases, posttraumatic osteoarthritis in 1 case, femoral head necrosis in 2 cases, osteoarthritis secondary to tuberculosis of the hip in 1 case, and osteoarthritis secondary to hip pyogenic infection in 1 case. In this operation, 23 hips had first revision, 4 had second revision, one had third revision, and one had fourth revision arthroplasties (Table 1). Before surgery, C-reactive protein, Interleukin-6 and erythrocyte sedimentation rate were obtained for every patient. If the infection can’t be ruled out, hip aspiration would be performed. If the acetabular component enters into the pelvis, the iliac artery angiography would be performed.

**Table 1:** Demographics of Patients

Parameters	Values
Gender (male/female)	13/15
Age (y)	56.4(36–75)
Body mass index (kg/m^2^)	23.9(18.3–29.6)
Side (R/L)	5/23
Diagnosis ^a^	
AL	25
PJI	3
Paprosky (IIIA/IIIB)	13/15

^a^*AL* Aseptic loosening, *PJI* Periprosthetic joint infection

### Surgical technique

The revision arthroplasty was performed by 5 senior surgeons in a laminar flow operating room. The posterolateral approach was used in all patients. First, the original acetabular component was exposed and removed. Curets, osteotomes, and hemispherical reamers were used to debride cement and scarred capsular tissue to fully expose the acetabulum and achieve a well-vascularized bone bed, at this point surgeon would assess whether hemispheric and augments or other materials can be used to complete the revision. In this step, attention should be paid to preserve the bone stock. At the same time, in other aseptic table, assistants started preparing the cryopreserved allogeneic cancellous bone which was previously stored at − 80 °C for at least 3 months. First, assistants soaked the bone in 5% povidone-iodine solution for 30 min and then made it into morselized bones with a diameter of about 0.5 cm–1 cm. Second, the bones were soaked in 5% povidone-iodine solution for 15 min again. Third, assistants washed the morselized bones with normal saline and then dipped them in 5 mg/ml vancomycin solution for 10 min. After that, the morselized bones were mixed with 500 mg vancomycin to spare. The amount of cancellous bone used from femoral heads or tibial plateau depends on the defect size. When the bone bed was ready, the morselized bones were filled into the cavitary defects and the surgeon reversely reamed them not very tightly. The flanges of the reconstruction cage (Zimmer Inc., Warsaw, IN, USA) were bent and shaped to fit the specific anatomy of the grafted acetabulum. The superior flange was fixed to the iliac bone with cancellous bone screws, and the inferior flanges were fixed to the ischium and the pubis. The cement was placed in the cage and pressed to make it exude on the edge of cage, which was to ensure that the cement penetrated uniformly into the gap between allograft bone bed and cage. Polyethylene cup was then cemented into the cage with an appropriate anteversion and abduction angle. Fifty-two mm (range 52–64) cages were most commonly used and the 28-mm metal femoral heads were used in all cases. Average of 7.2 (range 5–10) screws per cage were used.

### Postoperative management

All patients received cefuroxime and vancomycin to 3 days after operation. Enoxaparin was used to discharge followed by rivaroxaban for 3 weeks and pneumatic compression device was used to 24 h after operation. Patients began training the quadriceps femoris strength, hip flexion and hip abduction at the first day after operation and making touch-down weight bearing at two or 3 days after operation. Partial weight bearing began at 6 weeks after the operation and then transfers to full weight-bearing gradually. The patients were advised to avoid forced internal rotation and keep slight abduction with use of a wedged pillow for 3 months. Clinical and radiographic evaluation were performed at 3 months, 6 months, and 1 year after the operation, and then once a year until last follow-up.

Clinical assessment included Harris Hip Score (HHS) [25], Short Form-12 (SF-12) [26], and complications. For HHS, both pre and postoperative were obtained, 90 to 100 points were defined as “excellent”, 80 to 89 points were defined as “good”, 70 to 79 points were defined as “fair”, and lower than 70 points were defined as “poor”. For SF-12, physical and mental component were evaluated independently.

**Fig. 1:**
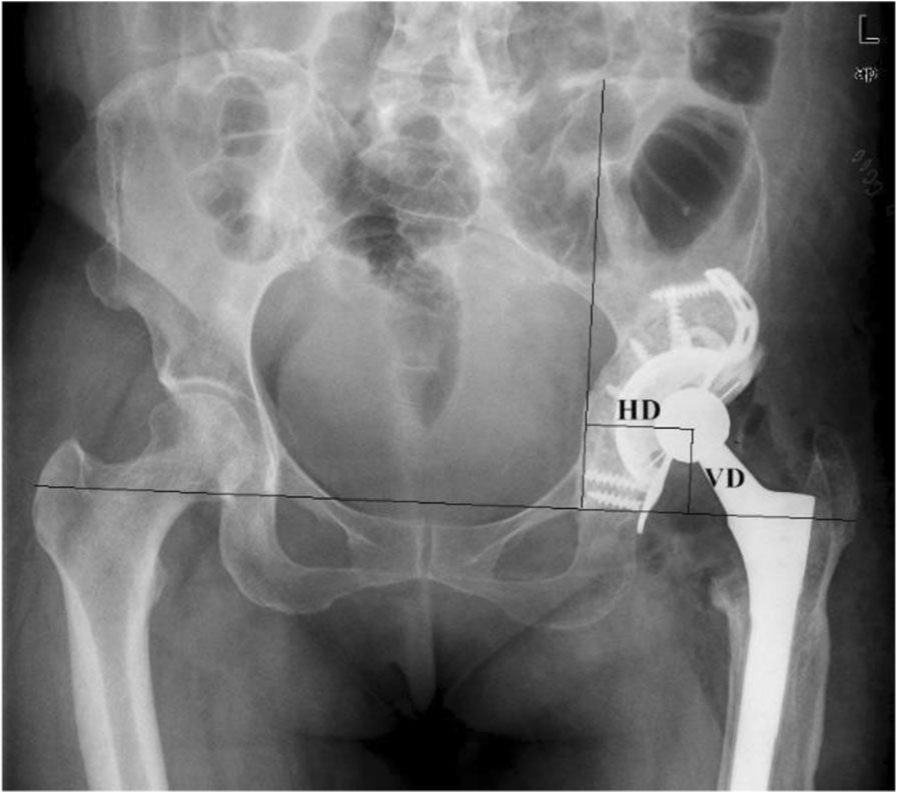
VD, vertical distance; HD, horizontal distance

Radiographic assessment was accomplished by taking standard anteroposterior radiographs of pelvis and anteroposterior and lateral radiographs of the hip at each follow-up of all patients, and if necessary, 3-dimensional computed tomography of the hip would be obtained. We measured the hip center of rotation in standard anteroposterior radiographs of pelvis [24]. The distance between femoral head center and reference line through the teardrop figure is defined as vertical distance (VD) and the distance between femoral head center and perpendicular reference line through the teardrop is defined as horizontal distance (HD), the changes of which is defined as vertical migration (VM) or horizontal migration (HM) (Fig. 1). According to the criteria of Gill et al. [27], more than 5 mm cage migration in the horizontal or vertical, screw breakage and progressive radiolucent lines present at the cage–bone interface medially and superiorly or around the screws were defined as loosening. As Gross et al. reported [28], incorporation of the allograft was defined radiologically by the presence of trabecular crossing the graft–host interface. The graft resorption was evaluated by anteroposterior radiographs and was graded as minor (<1/3 of graft resorbed), moderate (1/3 to 1/2 of graft resorbed), and severe (>1/2 of graft resorbed). We also described the graft resorption in the three zones of acetabulum defined by DeLee and Charnley [29].

### Statistical analysis

Quantitative data were presented as mean values ± standard deviation. Statistical package SPSS version 22 (SPSS version 22; IBM Corporation, USA) was used to perform statistical analyses. The pre and postoperative clinical and radiological data were compared using a paired Student’s t-test. *P* < 0.05 was considered to be statistically significant.

## Result

### Clinical results and complications

**Fig. 2:**
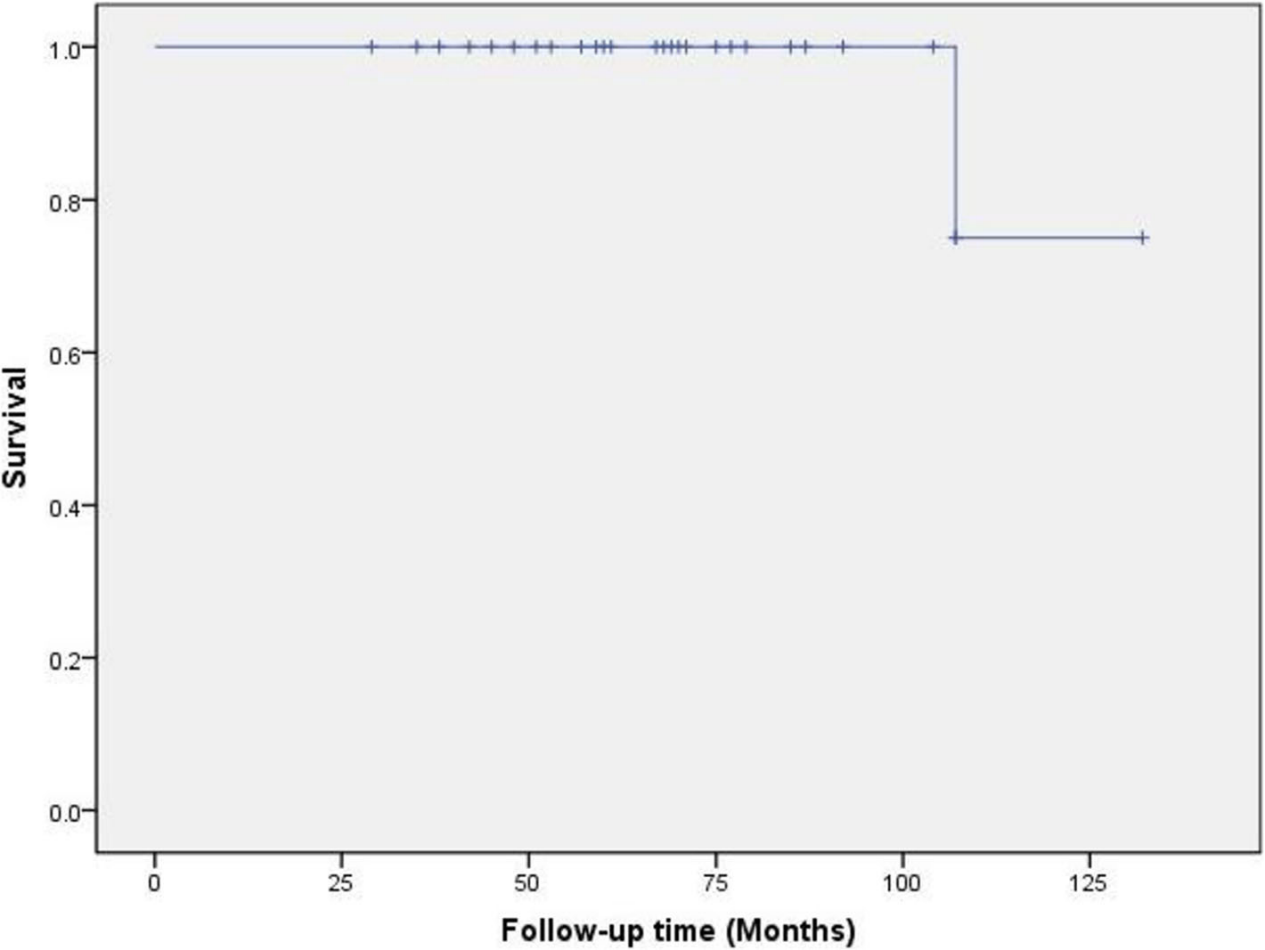
Kaplan-Meier reconstructional cage survivorship analysis with re-revision as the end point is shown

All patients were followed up with a mean follow-up of 79.5 months (range 38–141). We had 1 re-revision, the implant survival with acetabular re-revision as the end point is shown in Fig. 2. The HHS improved significantly from 31.4 (13–43) points preoperatively to 84.6 (55–94) points at the last follow-up (*p* < 0.01), in which 7 (25%) patients had an excellent score; 16 (57%) had a good score; 4 (14%) had a fair score; and 1 (4%) had a poor score (55 points) which was performed re-revision as mentioned later. Compared with preoperative, the SF-12 at the last follow-up has improved significantly (Table 2). There was 1 recurrent dislocation at 8 months after operation treated with plaster immobilization for 3 months with no re-dislocation afterwards and HHS of the patient was 85 points at the last follow-up, 1 sciatic nerve palsy recovered partially and the HHS of the patient was 72 points at the last follow-up, 1 acute renal injury was successfully treated, and 1 femoral prosthesis loosening at the 2nd years after operation treated with femur re-revision, whose acetabular prosthesis was stable and the HHS of the patient was 87 points at the last follow-up. There was no periprosthetic joint infection, no deep vein thrombosis, no vessel damage, and no complain about limbs length discrepancy (Table 3).

**Table 2:** Preoperative and postoperative comparison of Clinical and Radiological evaluation

Indicator	Preoperative	Postoperative	Last follow-up	*P* Value
HHS	31.4 ± 10.4		84.1 ± 7.8	.000
Rating (no. of hips)				
Excellent	0		7	
Good	0		16	
Fair	0		4	
poor	28		1	
SF-12				
Mental component	12.8 ± 3.1		23.9 ± 2.7	.000
Physical component	7.8 ± 1.3		21.3 ± 2.6	.000
Hip center (mm)				
Horizontal distance	42.1 ± 11.5	42.7 ± 6.2	43.1 ± 6.5	.773/.351^a^
Vertical distance	47.9 ± 17.2	22.3 ± 7.7	23.6 ± 9.2	.000/.012^a^
horizontal migration^b^			1.4 ± 1.7	
vertical migration^b^			1.3 ± 2.5	

^a^Preoperative versus postoperative/postoperative versus last follow-up. ^b^ Absolute value

**Table 3:** Post-operative complications

Age	Gender	Defect (paprosky)	Follow-up (month)	Complications	Procedure	Clinical Outcomes
45	M	IIIA	94	Recurrent dislocation	Plaster immobilization for 3 months	No re-dislocation
73	F	IIIA	53	Sciatic nerve palsy	Neuro nutrition drugs, Prednisolone and rehabilitation exercise	Partial recovery of sensory and motor function
72	F	IIIA	37	Acute renal injury	Supportive care	Fully recovered
42	M	IIIB (PD^a^)	42	Femoral prosthesis loosening at 2 years after operation; Radiolucency in the junction of DeLee and Charnley zone II and zone III	Revision of femoral prosthesis; conservative treatment for the radiolucency	Fracture healed, stable components; nonprogressive radiolucency
51	F	IIIB (PD^a^)	109	Aseptic loosening of the cage at 61 months after surgery	Re-revision with jumbo cup and tantalum augment	Stable components, well-functioning hip
62	F	IIIB	47	Radiolucency in the DeLee and Charnley zone III	Conservative treatment	Nonprogressive radiolucency

^a^Pelvic discontinuity

With acetabular components inserting into the pelvis, iliac angiography was performed on one of the patients and found that the acetabulum prosthesis compressed the internal iliac artery, so we performed internal iliac artery embolization before operation. The operation of the patient was smooth.

### Radiological results

**Fig. 3:**
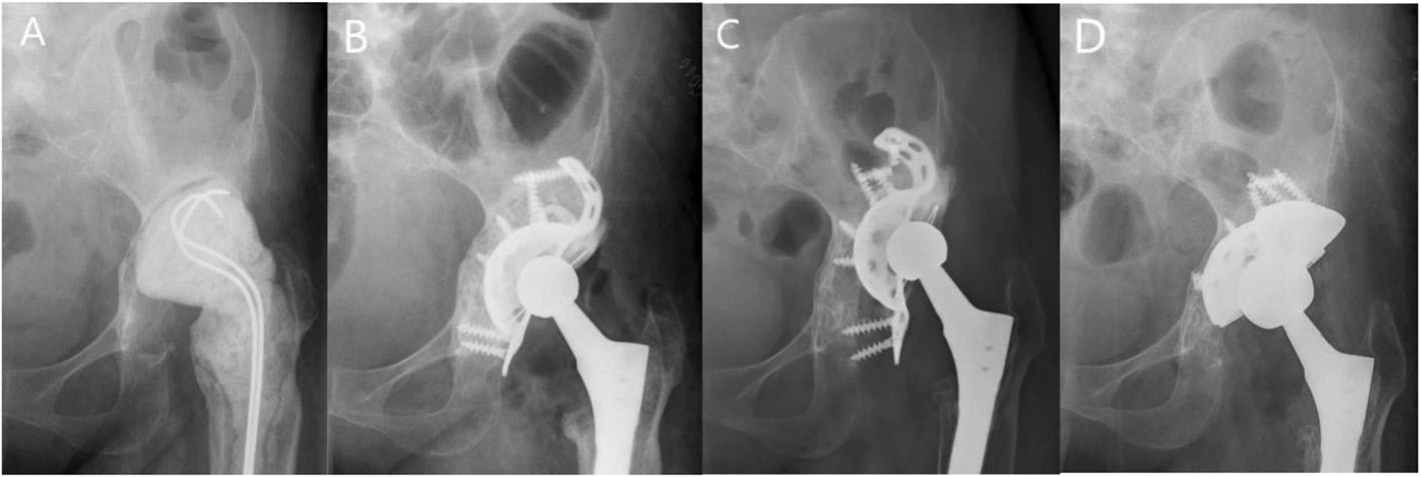
Radiographs of a 51-year-old woman with Paprosky IIIB acetabular bone defects and pelvic discontinuity was found intraoperatively. **a** Preoperative radiograph. **b** Immediate postoperative radiograph showed reconstruction cage and morselized allografts reconstructed the bone defect. **c** Radiograph at 61 months after revision suegery, the cage was loosening with one screw breakage. It was found intraoperatively that allograft was partially incorporated with the host bone and the pelvic discontinuity was healing. **d** Radiograph after re-revision with jumbo cup and tantalum augment

**Fig. 4:**
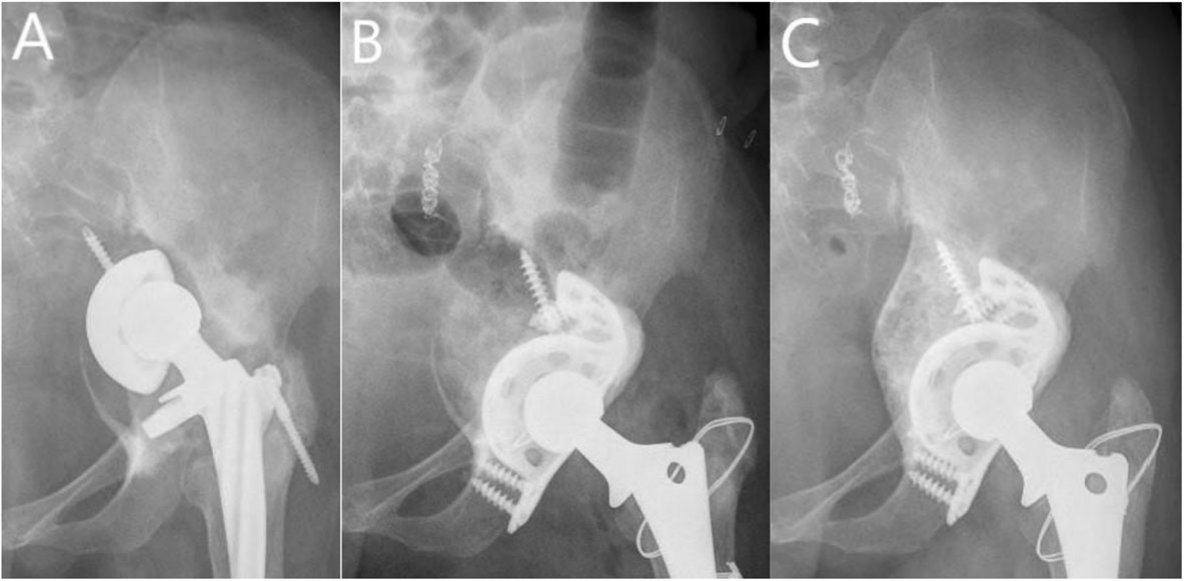
Radiographs of a 43-year-old woman with Paprosky IIIB acetabular bone defects and pelvic discontinuity. **a** Preoperative radiograph. **b** Immediate postoperative radiograph showed reconstruction cage and morselized allografts reconstructed the bone defect. **c** Radiograph at 96 months after revision suegery show that the cage remained stable and the allograft was completely incorporated with the host bone

As for the hip center of rotation, the horizontal distance was corrected from preoperative 42.1 mm (range 15.2–61.5) to postoperative 42.7 mm (range 34.3–53.5) (*P* > 0.05). The vertical distance was corrected from preoperative 47.9 mm (range 15.8–78.0) to postoperative 22.3 mm (range 12.2–40.5) (*P* < 0.05). There was only one (4%) patient with postoperative vertical distance more than 35 mm which was defined as a high hip center [30]. Comparing with 23 (82%) patients whose preoperative vertical distance was more than 35 mm, the hip center of rotation was improved obviously. There was no significant difference between postoperative and last follow-up horizontal distance (43.1 mm, range 34.1–56.9) (P > 0.05), and horizontal migration from postoperative to last follow-up was 1.4 mm (range 0.1–9.6). There was significant difference between postoperative and last follow-up vertical distances (23.6 mm, range12.4–54.3) (*P* < 0.05), and vertical migration from postoperative to last follow-up was 1.3 mm (range 0–13.8) (Table 2). Although the difference between postoperative and last follow-up vertical distances was significant, there was only 1 patient whose vertical migration (13.8 mm) and horizontal migration (9.6 mm) were both more than 5 mm at the last follow-up. And the cage of this patient was loosening with one screw breakage at 61 months after surgery, we performed acetabular re-revision surgery using jumbo cup and tantalum augment (Fig. 3). For 2 patients (7%), nonprogressive radiolucency appeared at the bone implant interface, the width of which was less than 2 mm. The radiolucency of 1 patient was in the junction of DeLee and Charnley zone II and zone III and the other patient was in DeLee and Charnley zone III. And according to Gross et al. [28], the resorption of these 2 patients was graded as minor. Complete incorporation was encountered in 25 patients (Fig. 4).

## Discussion

Severe acetabular bone defects and pelvic discontinuity are extremely complicated and challenging in revision arthroplasty. In this study, the acetabular bone defects of all patients were too large to treat with the combination of hemispheric cup and augments, therefore we used reconstructional cage and morselized allogeneic cancellous bone to reconstruct the bone defects. The mid-long term follow-up clinical and radiographic results is successful.

In our study, one patient (4%) with pelvic discontinuity suffered re-revision for the cage loosening with one screw breakage at 61 months after surgery, but we found that the allograft bone was partially incorporated with the host bone and the pelvic discontinuity was healing, and we performed the re-revision with jumbo cup and tantalum augment. Abolghasemian et al. [31] reported that they performed 50 hips acetabular re-revision surgery who used structural or morselized allograft bone with a cage or ring for previous revision, and they found that a simple revision without using allograft, augments, rings or cages could be performed in 31 (62%) hips and 17 hips (34%) owing to the restoration of bone stock. In prior review by Baauw et al. [32], the average re-revision rate of large acetabular defects revised with antiprotrusio cage in 8 studies including 315 hips is 3.5%, which is similar to the results of this study.

In revision arthroplasty, the use of allografts is related to a high risk for infection, which remains as an important cause of reoperation [9, 18, 19, 32–34]. According to Aponte-Tinao et al. [35], their average 106 months follow-up of 673 patients using massive bone allografts showed that 60 patients (9%) had a bacterial infection of the allograft. The allograft we used was from the bone bank of our province and was stored at − 80 °C, we sterilized it with povidone-iodine and vancomycin solution before surgery. It is well-known that polyvidone has excellent antimicrobial efficacy to gram-positive bacteria, fungi, and bacterial endospores. Polyvidone was widely used to sterilize the dropped bone graft, Bauer et al. [21] and Soyer et al. [22] showed that 10% povidone-iodine not only had good sterilization ability but also had a relative advantage in maintaining the tissue viability of the bone. In recent years, the animal testing of Jiang et al. [23] and Zhao et al. [20] found that povidone-iodine could promote osteogenesis and could protect the properties of allogenic bone compared with commonly used irradiation. Buttaro et al. [36] used cancellous bone allograft with vancomycin in revision hip arthroplasty and they found that this method did not affect allograft incorporation, had no nephrotoxicity and seemed to be beneficial by preventing infection. In our study, there was no infection, but we should consider that we had a smaller sample size. In summary, according to our follow-up results, the method we used to sterilize allograft has a simple procedure, don’t affect bone incorporation, and has a good disinfection effect.

In this series, we just used reamer reversely reamed the morselized bone intraoperatively, which is similar to Ding et al. [17]. In their series, 29 hips with an average follow-up of 73 months had a good mid-term outcome with no patient need re-revision and 23 hips achieved complete incorporation. And in our study, complete incorporation was encountered in 25 patients (89%), which achieved good outcome and seems better than Ding (79%) [17]. In previous studies, mid-term result of impaction bone grafting combining with cage showed the failure rate was 0–16% [8, 33, 37]. Recently, Akel et al. reported a long-term result of this combination, the failure rate was 8.1% [38]. Although the results of these studies were good, the results mixed Paprosky type II and III bone defects. Our study shows that reconstructional cage and morselized allografts is also a good option for Paprosky type III bone defects in acetabular revision.

In this series, one patient had experienced sciatic nerve palsy after the surgery, which gradually recovered by using an ankle brace. In some prior studies [9, 12, 13, 18], this complication was also reported with an incidence lower than 3%. However, in complicated acetabular revision, it seems impossible to completely avoid sciatic nerve palsy. Limiting dissecting posterior and inferior soft tissue of ischium may be conducive to reduce possibility of postoperative sciatic nerve palsy. Also, there was 1 recurrent dislocation in this series. Winter et al. [37] summarized that using lateral approach and strictly arranging rehabilitation plan may attribute to reduce dislocation rate and we think that appropriate anteversion and abduction angle of the cup is also important.

As a retrospective and observational study, there are several limitations. First, like most of the previous studies, this study lacks a control group using different devices, so we can just draw a general conclusion by comparing with previous similar studies. Second, we assess the allograft incorporation by radiograph which unable to display the central part of the allograft. The developing technique of reducing metal artifact CT may be useful. Third, our study is a mid-long term follow-up; and longer term follow-up is required to evaluate the outcome. But to our best knowledge, the clinical follow-up outcome of the way we sterilize bone allograft is barely reported.

## Conclusion

In this study, the reconstructional cage combining with morselized allografts without impaction achieves a good result with a high complete allograft incorporation rate in Paprosky type III acetabular bone defects, which restores acetabular bone stock and may be beneficial to further revision. But in patients with pelvic discontinuity, this method should be used cautiously. Our mid-term follow-up outcome indicates that sterilizing bone allograft with povidone-iodine and vancomycin is a simple and effective way, but long-term follow-up and large sample studies are required to further evaluate the efficiency.

## Data Availability

The datasets used and/or analyzed during the current study are available from the corresponding author on reasonable request.

## Abbreviations

HHS: Harris Hip Score
SF-12: 12-item short-form health survey questionnaire
